# Deep learning-assisted ultrathin broadband vanadium dioxide-based polarization-insensitive terahertz metamaterial absorber

**DOI:** 10.1038/s41598-026-50729-2

**Published:** 2026-04-26

**Authors:** Pujita Bhatt, Prince Jain, Anand Joshi

**Affiliations:** 1 Department of Mechatronics Engineering, Parul Institute of Technology, Parul University, Vadodara, 391760 Gujarat India; 2 Micro Nano Research & Development Center, Parul University, Vadodara, 391760 Gujarat India; 3 Department of Mechanical Engineering, Parul Institute of Engineering & Technology, Parul University, Vadodara, 391760 Gujarat India

**Keywords:** Broadband absorption, Deep learning, Metamaterial, Multi-layer perceptron, Terahertz, Vanadium dioxide, Engineering, Materials science, Optics and photonics, Physics

## Abstract

In this article, we presented a broadband absorber structure design consist of double arrays of VO_2_ based complementary plus placed on top of a polyimide dielectric spacer. The absorber exhibits a compact design and ultrathin thickness of 0.328 λ and 0.062 λ, respectively, and achieves absorptivity above 90% over the 2.66–6.48 THz range, corresponding to a relative bandwidth of 83.6%. The physical mechanism of broadband absorption is explained by parametric analysis with electric, magnetic field, and surface current distributions. Furthermore, the proposed absorber has wide incident angle absorption and polarization insensitivity due to its symmetrical structure. To optimize the absorption performance, Deep learning techniques such as Multi-Layer Perceptron (MLP), Residual MLP, and Deep & Cross Network (DCN) models are introduced. Prediction performance is evaluated using 5- and 10-fold cross-validation, with the Residual MLP achieving the highest R^2^ and lowest error metrics. The proposed broadband absorber with deep learning has potential applications in electromagnetic stealth, cloaking, modulators, detection, sensing, and energy harvesting.

## Introduction

Metamaterials are artificially engineered structures that exhibit unconventional EM characteristics such as negative refractive index, allowing applications in absorbers, stealth technology, imaging, modulation, and cloaking^1–3^. In the terahertz (THz) regime, metamaterial absorbers (MMAs) have gained significant attention due to their ability to achieve strong EM control at subwavelength scales^4,5^. The majority of early THz MMAs had a narrow bandwidth due to strong EM resonance at a specific frequency, limiting their practical applications. Two conventional approaches are commonly used to enhance absorption bandwidth: stacking metal-dielectric layers^6^, and designing coplanar configurations composed of multiple resonators with different dimensions^7,8^. However, the first method has manufacturing issues, whereas the second method has a large unit cell when developing miniaturised absorbers. As a result, significant effort has been made to develop broadband MMAs with simple resonant structures^9,10^.

To address the growing demand for broadband and dynamically tunable THz absorbers, vanadium dioxide (VO_2_) has become a widely used active material because its insulator-metal transition can be induced electrically, thermally, or optically^11–13^. This transition enables large conductivity modulation and perfect absorption control in THz devices^14^. Liu et al. demonstrated a VO_2_ based hybrid MMA that transitions from low absorption (~ 3%) to broadband absorption exceeding 90%, achieving a relative bandwidth of approximately 83%^15^. Similarly, Yan et al. proposed a VO_2_ based switchable terahertz metasurface that enables broadband absorption above 90% over a 75% relative bandwidth, along with reconfigurable polarization conversion^16^. These studies highlight the suitability of VO_2_ for tunable THz absorbers, where its reversible conductivity change enables dynamic adjustment of resonance behavior and absorption intensity without requiring any geometric change. Despite these advances, MMA design and optimization are performed using CST simulation software involved iterative parameter sweeps, which makes the process computationally expensive and time-intensive^17^.

Recently, deep learning (DL) and machine learning (ML) methods have been increasingly adopted to overcome these limitations by enabling fast and accurate prediction of EM responses of MMAs. Bhatt et al. employed various ML models, including CatBoost, ExtraTrees, and KNN, to predict and optimize the multi-band absorption characteristics of a terahertz MMA^18^. Patel et al. integrated a ML based optimization framework to accelerate the design of a hash-shaped solar MMA reducing simulation time by approximately 75%^19^. Mahesh et al. introduced a deep convolutional autoencoder–based algorithm for the inverse design of a low-frequency acoustic absorber^20^. Mahesh et al. developed a deep neural network–driven inverse prediction framework to determine the geometric parameters of a Helmholtz resonator–based acoustic absorber aimed at efficient low-frequency absorption^21^. These findings demonstrate the potential of ML and DL-assisted methods as efficient and reliable tools for designing MMA.

**Fig. 1:**
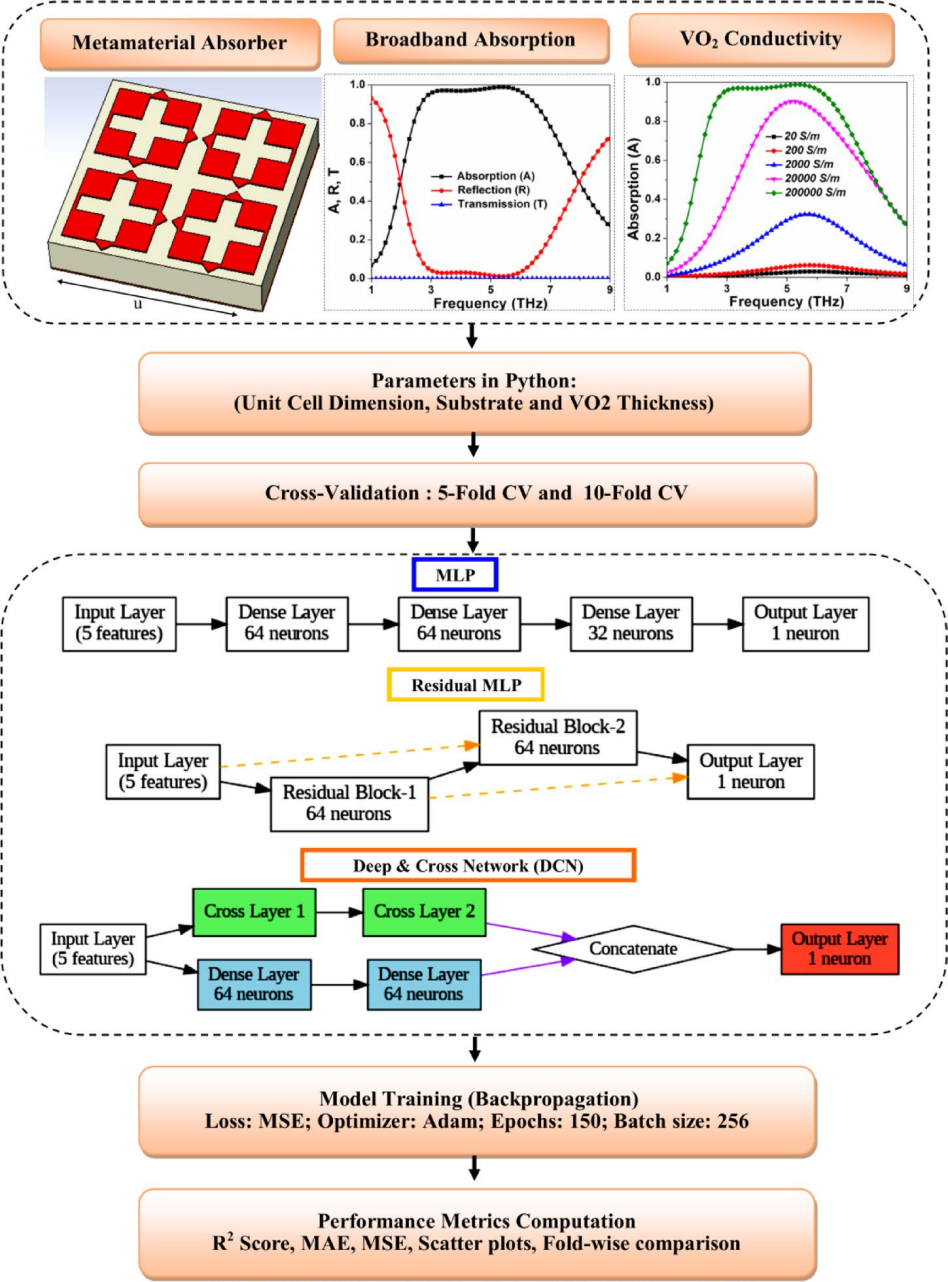
Workflow of the deep learning framework for proposed metamaterial absorber.

This papers presents a complementary plus-shaped MMA is proposed using a simulation-to-modeling workflow, as illustrated in Fig. 1. The absorber achieves broadband absorption from 2.66 to 6.48 THz, corresponding to an effective bandwidth of 3.82 THz. To reduce the computational cost associated with repeated full-wave simulations, extracted geometric and material parameters are used to develop data-driven regression models. Multi-Layer Perceptron (MLP), Residual MLP (R-MLP), and Deep & Cross Network (DCN) methods are employed to learn the nonlinear mapping between frequency, structural parameters, and absorptivity. Model robustness is validated using 5-fold and 10-fold cross-validation, while prediction accuracy is evaluated through R^2^, MAE, and MSE metrics, supported by scatter and residual analyses.

## Absorber design

**Fig. 2:**
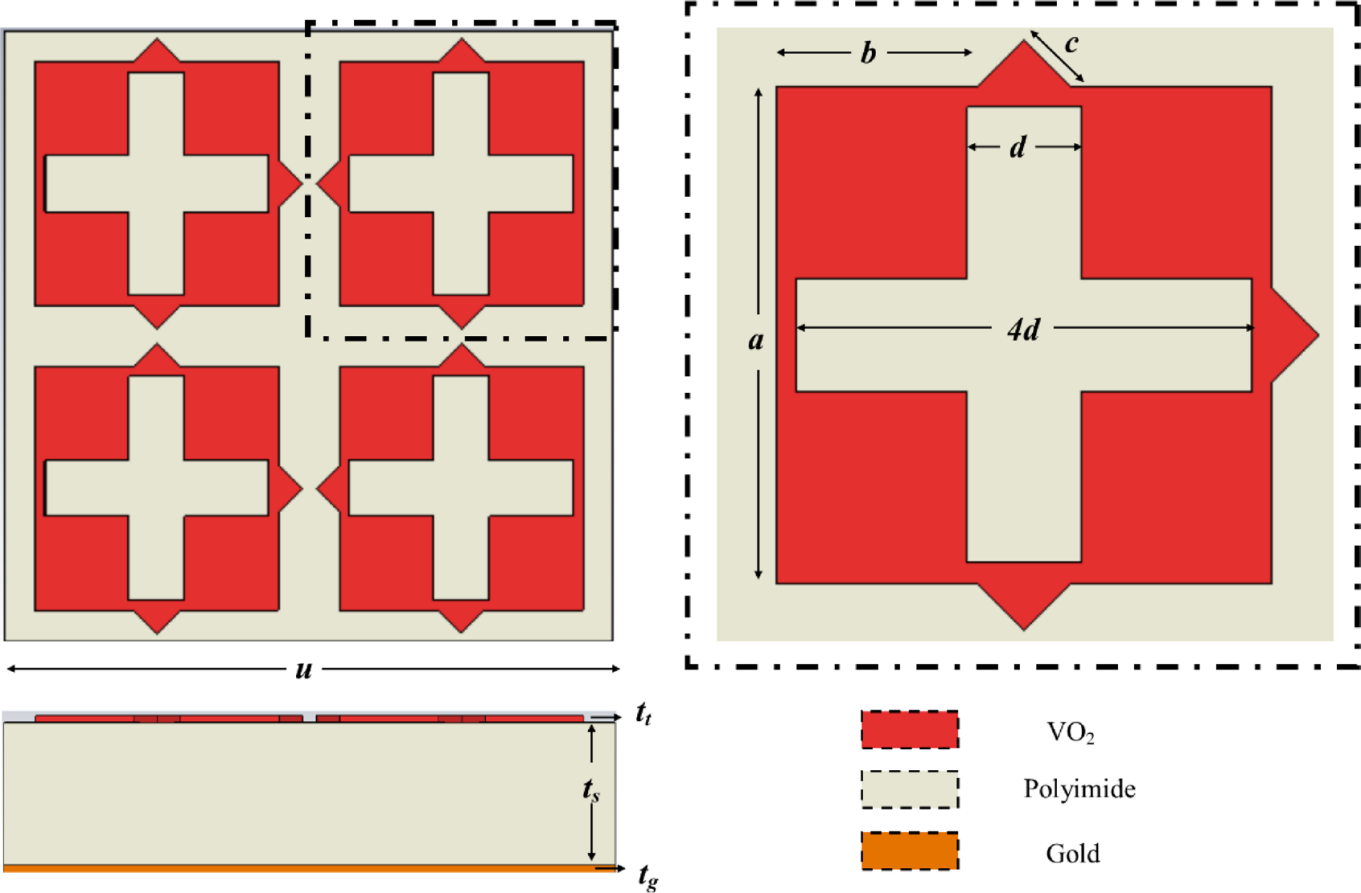
Schematic representation of the proposed MMA with its enlarged and side view.

Figure 2 illustrates the geometric configuration of the proposed broadband MMA, along with an enlarged view of the single resonator. The absorber is realized as a periodic array in which the effective unit cell consists of a 2 × 2 arrangement of identical plus-shaped resonators. The geometric parameters listed in Table 1 were determined through parametric analysis within practical ranges to study their influence on absorption performance. The proposed three-layer configuration, comprising the VO_2_ resonant layer, a dielectric spacer, and a metallic ground plane, enables broadband absorption through resonant coupling and effective impedance matching. In the terahertz design, the dielectric response of the VO_2_ film^22^ is demonstrated using the Drude formulation, as given in Eq. (1):

1 $$\:{\epsilon\:}_{{VO}_{2}}\left(\omega\:\right)=\:{\epsilon\:}_{\infty\:}-\frac{\sigma\:{\omega\:}_{p}^{2}\left({\sigma\:}_{0}\right)}{{\sigma\:}_{0}({\omega\:}^{2}+i\gamma\:\omega\:)}$$

where $\:{\epsilon\:}_{\infty\:}$ = 12 represents the high-frequency permittivity, $\:\gamma\:$ = 5.73 × 10^13^ rad/s is the collision frequency, $\:{\sigma\:}_{0}$ = 3 × 10^5^ S/m is the reference conductivity and $\:{\omega\:}_{p}$ = 1.4 × 10^15^ rad/s denotes the plasma frequency. This study includes where VO_2_ acts as metallic when its conductivity $\:{\sigma\:}_{{VO}_{2}}$ is set to 2 × 10^5^ S/m whereas it acts as the insulator at lower conductivity 20 S/m. Since VO_2_ conductivity is temperature-dependent, this parameter can be tuned thermally. Below the transition temperature (~ 340 K), VO_2_ exhibits a monoclinic crystal structure associated with a semiconducting state characterized by high resistivity and infrared transmission.

In the numerical simulations, periodic unit-cell boundary conditions are applied along the x and y directions, while an open (add space) boundary is assigned along the z axis to emulate free-space wave propagation. The incident EM wave is configured such that the electric field (E) and magnetic field (H) lie in the plane of incidence, and the propagation vector (*k*) is oriented normal to the surface of the metamaterial absorber. The reflection coefficient *S*_11_ and transmission coefficient *S*_21_ are extracted through S-parameter analysis^23^. The absorptance is then determined using $\:A=1-{\left|{S}_{11}\right|}^{2}-{\left|{S}_{21}\right|}^{2}$. Because the bottom layer is a continuous gold film, transmission is completely suppressed, resulting in *S*_21_=0^24^. The effective impedance of the absorber can be matched to that of free space by adjusting the structural characteristics, which lowers reflection and increases absorption.

**Table 1:** Dimensions of the terahertz absorber.

Parameters	Values(µm)	Description
*u*	37	Unit cell dimension
*a*	14.80	Squared side length
*b*	5.99	Offset distance
*c*	2	Triangle tip length
*d*	3.4	Cross arm width
*t*_s_	6.5	Substrate thickness
*t*_g_	0.5	Ground layer thickness
*t*_t_	0.3	Resonator (VO_2_) thickness

**Fig. 3:**
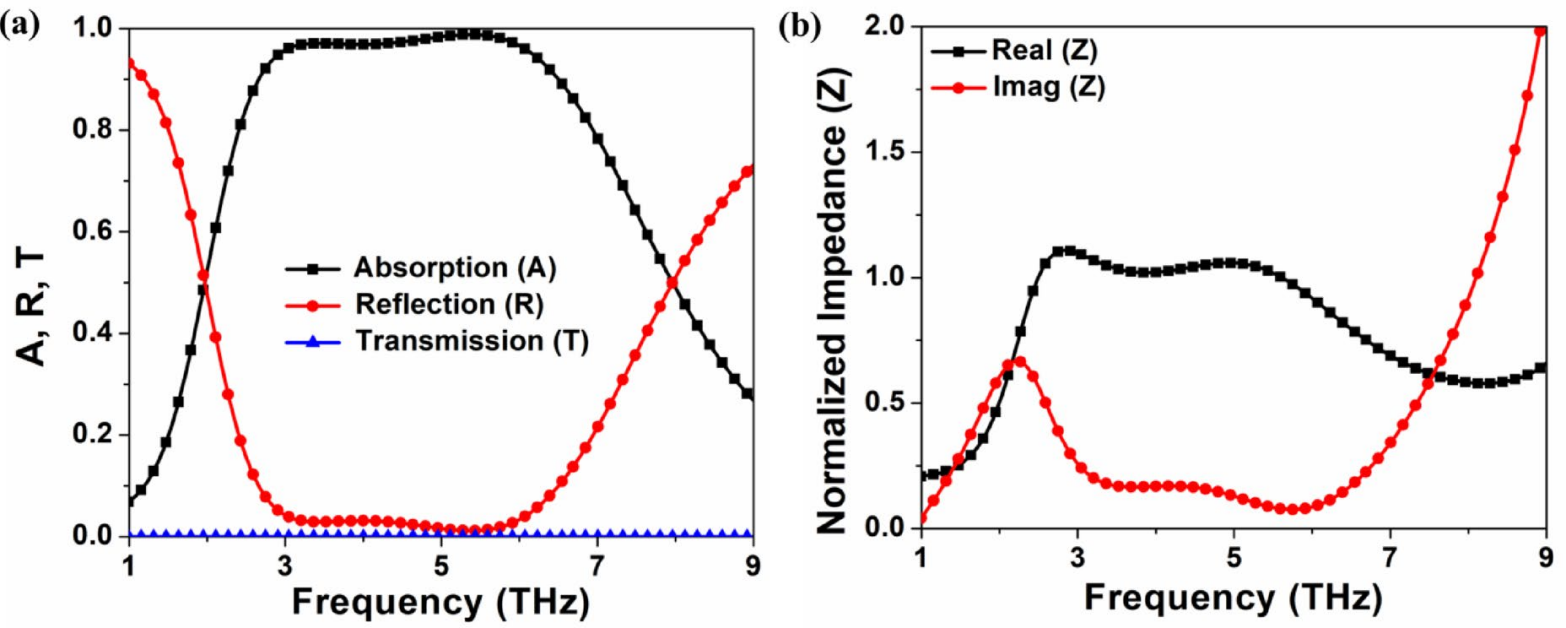
(**a**) Simulated reflection, absorption, and transmission spectra and (**b**) Corresponding normalized real and imaginary impedance of the proposed absorber.

Figure 3a shows that absorption approaches unity in the broadband range while reflection is limited and transmission is almost zero, confirming near-perfect absorber behavior with the dimensions provided in Table 1. In particular, the absorption exceeds 90% over the 2.66–6.48 THz range, corresponding to an effective bandwidth of 3.82 THz. In Fig. 3b, the real part of the normalized impedance (Re (Z)) remains close to unity across the operating band and the imaginary part (Im (Z)) stays near zero, indicating impedance matching with free space and validating the resonance-driven suppression of Fresnel reflection.

**Fig. 4:**
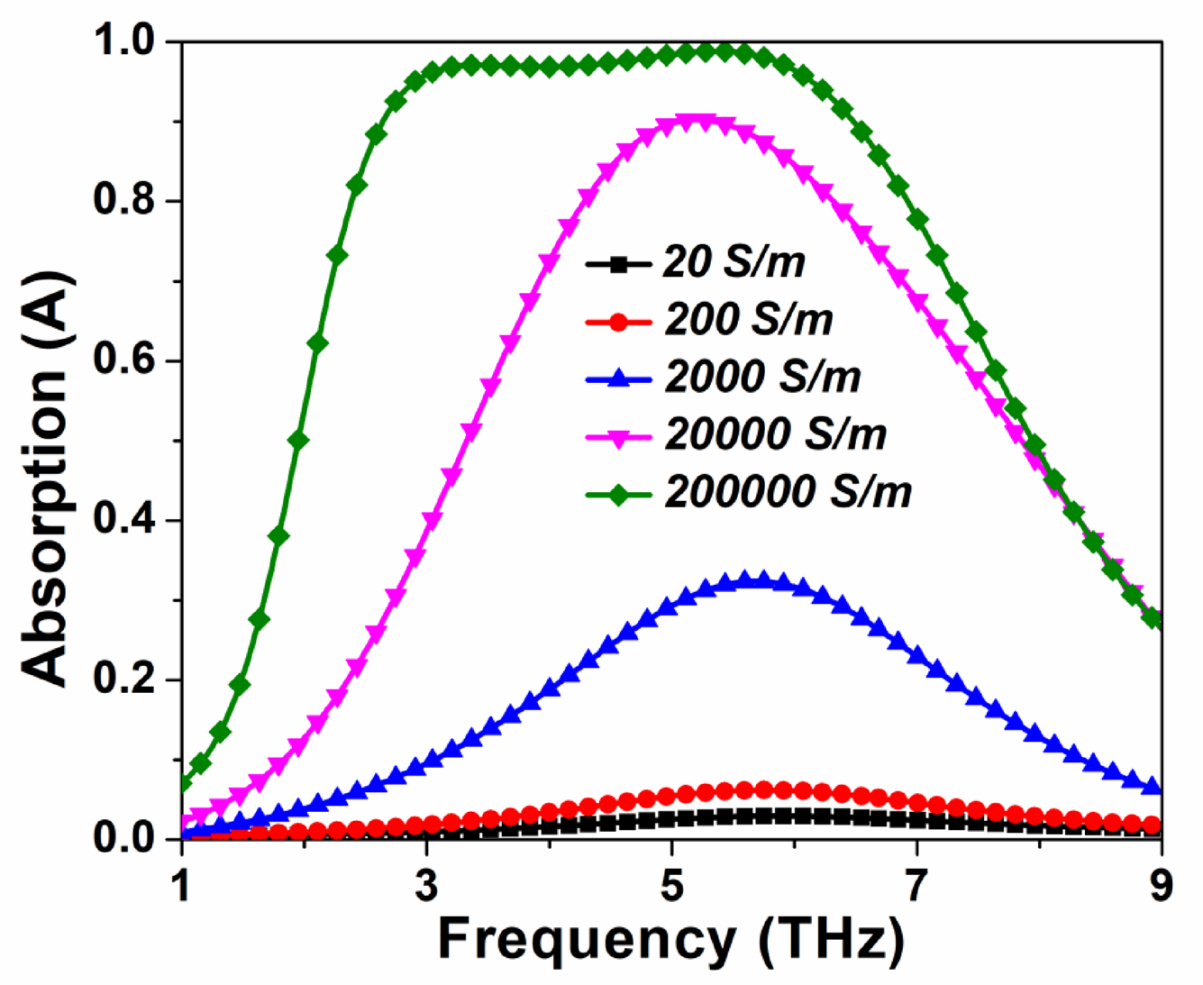
Absorption response for different VO_2_ conductivities.

**Fig. 5:**
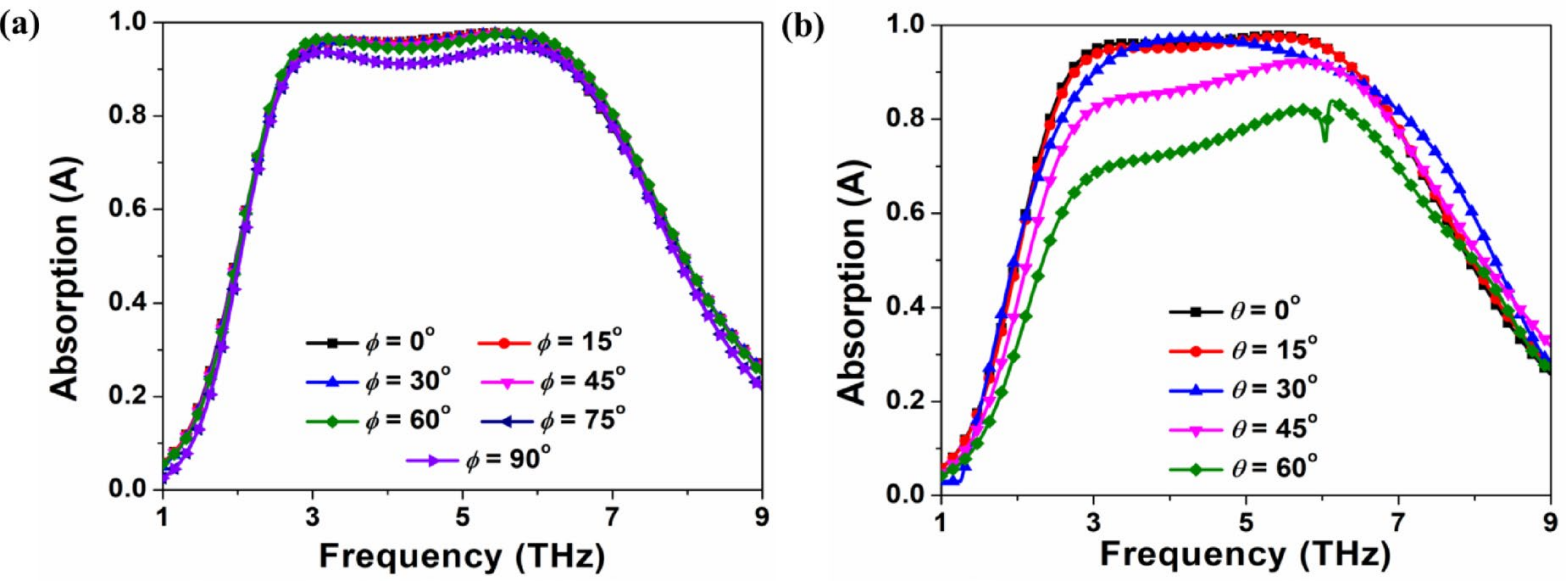
Simulated absorption response for different values of (**a**) polarization angle (*ϕ*) and (**b**) incident angle (*θ*).

Figure 4 illustrate the influence of VO_2_ conductivity on the absorption response. At low conductivities, VO_2_ exhibits an insulating state with weak plasmonic interaction and poor surface current formation, resulting in negligible absorption. As the conductivity increases to 200,000 S/m, it allows for surface current excitation, enhanced electric and magnetic resonant coupling, and near-unity broadband absorption. In Fig. 5a, the absorption spectra remain same for polarization angles (*ϕ*) from 0° to 90° because of the symmetric structure of the MMA which confirms the polarization-insensitive behavior. In Fig. 5b, the absorber maintains high absorption levels for incident angles *θ* = 0° to 45° under transverse electric (TE) polarization, confirming electric–magnetic mode coupling.

**Fig. 6:**
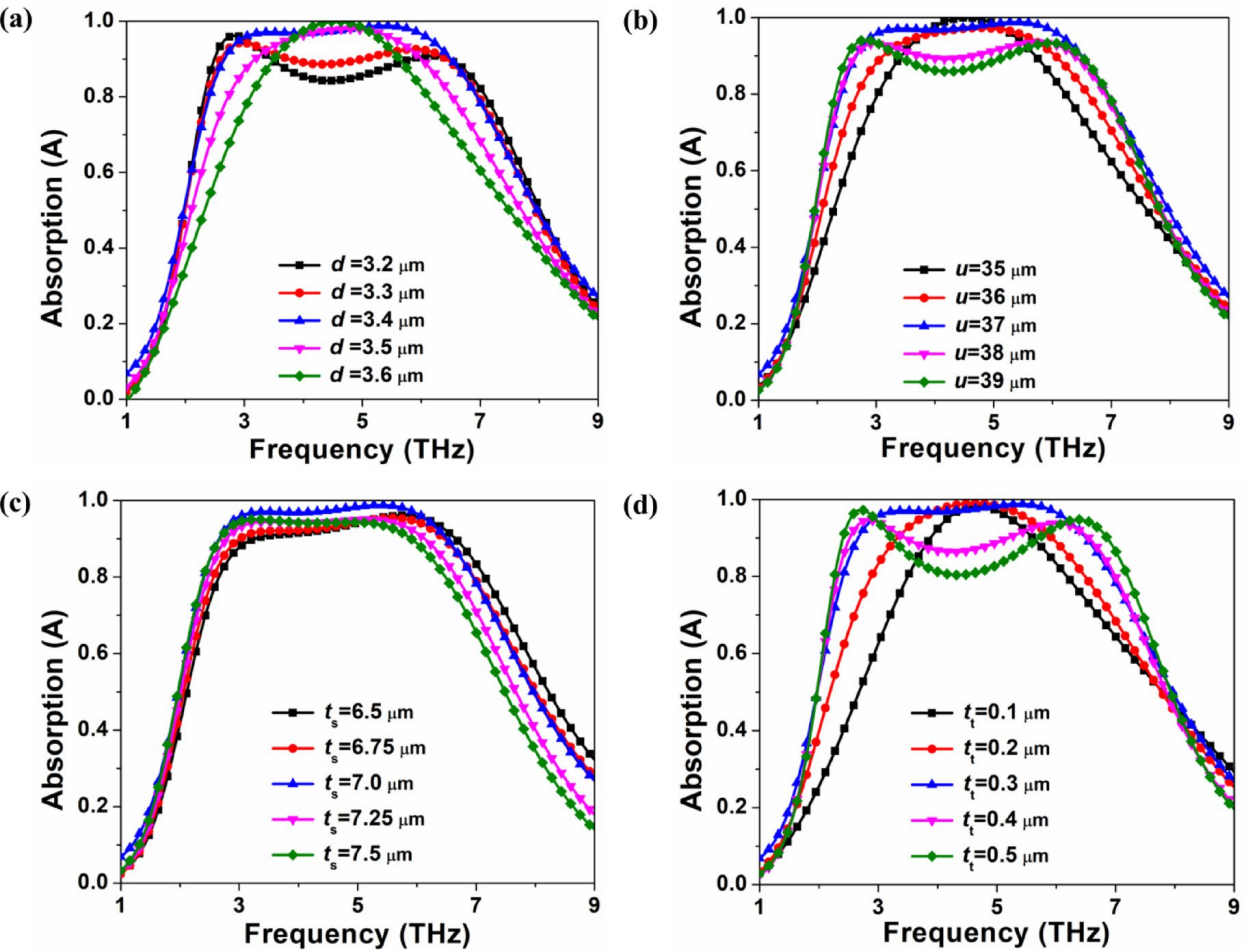
Absorption response for variations in (**a**) cross-arm width, (**b**) unit-cell period, (**c**) substrate thickness and (**d**) VO_2_ thickness.

Figure 6 presents the absorption spectra with variations in resonator width, unit-cell periodicity, dielectric spacer thickness, and resonator thickness. These parameters are the dominant geometric factors that influence resonance position, bandwidth, impedance matching, and absorption peak. In Fig. 6a, varying the cross-arm width (*d*) from 3.2 to 3.6 μm alters the effective capacitance of the resonator, resulting in a slight resonance shift while maintaining broadband absorption above 90%. Changing the unit-cell periodicity (u) from 35 to 39 μm impacts inter-cell EM coupling, resulting in slight resonance dispersion without considerable change in absorption bandwidth, as illustrated in Fig. 6b. Figure 6c shows how changing the substrate thickness (*t*_s_) from 6.5 to 7.5 μm affects the magnetic dipole confinement between the resonator and ground plane. An optimal *t*_s_ ensures enhanced field trapping and reduced reflection, thereby maintaining high absorptivity. As shown in Fig. 6d, variation of the VO_2_ thickness (*t*_t_) from 0.1 to 0.5 μm affects the plasmonic interaction strength, resulting in a modest reduction in absorption with increased thickness.

**Fig. 7:**
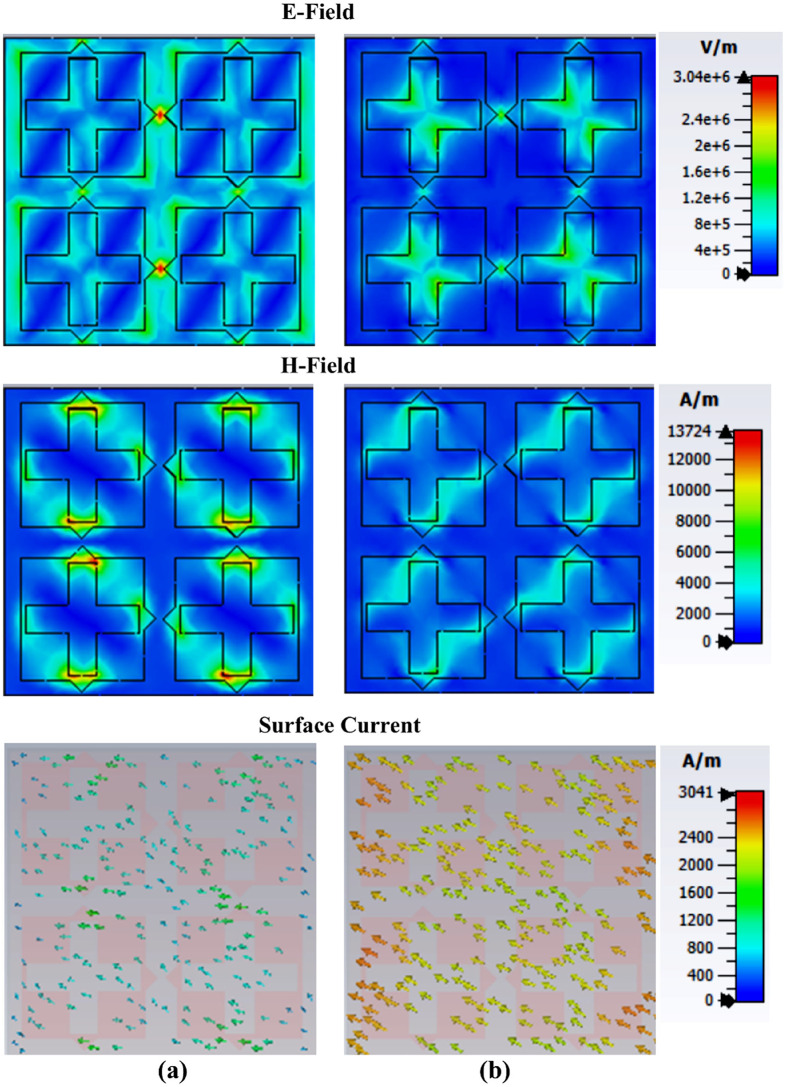
(a) Electric (E) field, (b) magnetic (H) field, and (c) surface current distributions at 3.04 THz (Left side) and 6.67 THz (Right side).

EM field and surface current distributions clarify the resonance mechanisms responsible for perfect absorption in the proposed MMA, as shown in Fig. 7. The E-field is concentrated around the inner arm junctions and the tapered ends of the complementary plus-shaped resonator, indicating an electric dipole response at the lower resonance of 3.04 THz, as shown in Fig. 7(a). The corresponding H-field exhibits circulating loops confined within the resonator gaps, confirming the excitation of magnetic resonance supported by antiparallel surface currents along opposite arms, as illustrated in Fig. 7(b) and Fig. 7(c). At the higher resonance of 6.67 THz, the E-field becomes more broadly distributed around the resonator perimeter and diamond-shaped boundaries, while the H-field exhibits extended circulation paths. The surface current density becomes more distributed, forming multiple current loops and hybridized higher-order resonant circuits, as shown in Fig. 7(c). These EM field patterns confirm both fundamental dipolar resonance and higher-order hybridized modes, enabling wideband absorption.

Table 2 shows the comparative analysis of the proposed absorber against state-of-the-art terahertz absorbers in terms of electrical size, bandwidth, structural complexity, and machine-learning integration. The graphene maze absorber in^25^ requires a large period of 0.82 λ and a thickness of 0.15 λ, while the Oblique Split-Ring Resonator (OSRR) design in^15^ has a moderate period (0.277 λ) but still a thickness of 0.083 λ. Multi-layer hybrid structures in^26^ and^16^ use six layers, increasing fabrication complexity despite keeping thickness near 0.13 λ. The square-loop design in^27^ uses a small thickness (0.089 λ) but exhibits one of the largest electrical periods (0.558 λ). The graphene cross-ring structure in^28^ shows both a large period (0.43 λ) and thicker profile (0.158 λ). Likewise, the hybrid concentric-ring absorber in^29^ requires six layers with thickness around 0.13 λ. In contrast, this work achieves one of the smallest electrical thicknesses (0.062 λ) and a compact period (0.328 λ) using only three layers, while maintaining a wide Relative Absorption Bandwidth (RAB) of 83.6%.

**Table 2:** Comparison with reported MMAs with the proposed THz MMA.

Ref	Structure	Top layer	Layers	Absorption range	Periodicity and thickness	RAB (%)	Absorption (%)	ML integration
This work	Complementary plus shaped	VO_2_	3	2.66–6.48 THz	0.328 λ and 0.062 λ	83.6%	> 90%	Yes
^25^	Complementary Interdigitated Maze Shape	Graphene	3	3.7-8 THz	0.82 λ and 0.15 λ	73.5%	> 90%	No
^15^	OSRR	VO_2_	3	0.7–1.7 THz	0.277 λ and 0.083 λ	83%	> 90%	No
^26^	Six-petal resonator (Top) + Three-sector curved resonator (Middle)	VO_2_ (Top) + Gold (Middle)	6	1.36–3.38 THz	0.18 λ and 0.13 λ	85.2%	> 90%	No
^30^	Cross-shaped and four L-shaped	VO_2_	3	3.25–7.35 THz	0.26 λ and 0.070 λ	77.4%	> 90%	No
^16^	Square Complementary Ring (Top) + U-Resonator Hybrid (Middle)	VO_2_ (Top) + Gold (Middle)	6	0.74–1.62 THz	0.123 λ and 0.121 λ	74.6%	> 90%	No
^27^	Square loops	VO_2_	3	1.85–4.3 THz	0.558 λ and 0.089 λ	79.7%	> 90%	No
^28^	Cross-Ring Resonator	Graphene	3	3–6 THz	0.43 λ and 0.158 λ	66.7%	> 90%	No
^29^	Hybrid concentric-ring (Top) + split-ring arc structure (Middle)	VO_2_ (Top) + Gold (Middle)	6	1.485–3.575 THz	0.20 λ and 0.13 λ	82.6	> 90%	No
^31^	Conference-matrix resonator	Ni	3	250–1150 nm	0.67 λ and 0.45 λ	128.6	> 99%	Yes
^32^	Ring–bar resonator	Gold	3	2.93, 3.88, 4.30, 5.43 THz	0.20 λ and 0.048 λ	-	> 99%	Yes
^18^	Dual octagonal rings with peripheral strips	Gold	3	2.64, 4.21, 5.43, 7.9, 8.5 THz	0.255 λ and 0.033 λ	-	> 97%	Yes

**Fig. 8:**
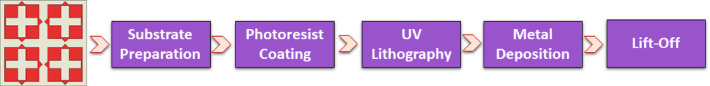
Fabrication workflow for the proposed metamaterial absorber.

The proposed metamaterial absorber is fabricated through a sequence of precise fabrication steps as shown in Fig. 8. The process starts with designing a periodic cross-shaped VO_2_ resonator array, followed by preparing a clean SiO_2_ substrate. Alignment marks are patterned for accurate mask positioning, after which a photoresist layer is spin-coated to define the VO_2_ structures. After development, a thin VO_2_ film is deposited using pulsed-laser deposition or RF sputtering and then annealed to achieve the required phase-transition properties. A lift-off process removes excess resist, leaving well-defined VO_2_ resonators on the substrate. The fabricated structures are inspected microscopically to confirm pattern accuracy and uniformity, resulting in a tunable, phase change based MMA ready for characterization^33^.

## Deep learning analysis

In addition to the electromagnetic analysis, deep learning models were developed to predict the absorptivity response of the proposed structure from its geometric parameters, as shown in Fig. 1. The dataset was prepared using unit-cell dimension, plus-shaped arm width, substrate thickness, VO_2_ thickness, and operating frequency, as well as the corresponding absorptivity values. Three DL methods are employed to learn the nonlinear relationship between the metamaterial geometry and its absorption behavior. The first model is a multilayer perceptron, which serves as a baseline. It is made up of stacked fully connected layers with nonlinear activation functions that can capture basic to moderately complex dependencies between input parameters^34,35^. While this architecture is useful for simple mappings, it has limitations when dealing with higher-order interactions and coupled effects that are common in metamaterial responses. To address these challenges, Residual MLP is adopted by incorporating skip connections between successive layers, which allow information to propagate more efficiently through the network^36,37^. Deep & Cross Network is specifically designed to simulate explicit interactions between input features while also learning complex nonlinear representations. The DCN combines cross layers that produce multiplicative feature interactions with a deep neural branch that performs hierarchical feature learning^38^. This enables efficient exploration and refinement of the absorber design across the parameter space.

To obtain reliable performance estimates and reduce sensitivity to a single data split, both 5-fold and 10-fold cross-validation (CV) are employed. In each cross-validation cycle, the dataset is divided into mutually exclusive folds, with the model trained on k-1 folds and tested on the remaining fold^39,40^. This procedure is repeated until every fold has been used once for validation. For each network architecture and validation strategy, predictions from all folds are combined into a single predicted-versus-actual scatter distribution. Once trained on CST-generated data, the deep learning models can estimate absorptivity for new geometric and frequency combinations without running additional full-wave simulations. This makes it easier to examine the design space and adjust parameters such as the unit-cell period, arm width, substrate thickness, and VO_2_ layer to meet specific requirements.

**Fig. 9:**
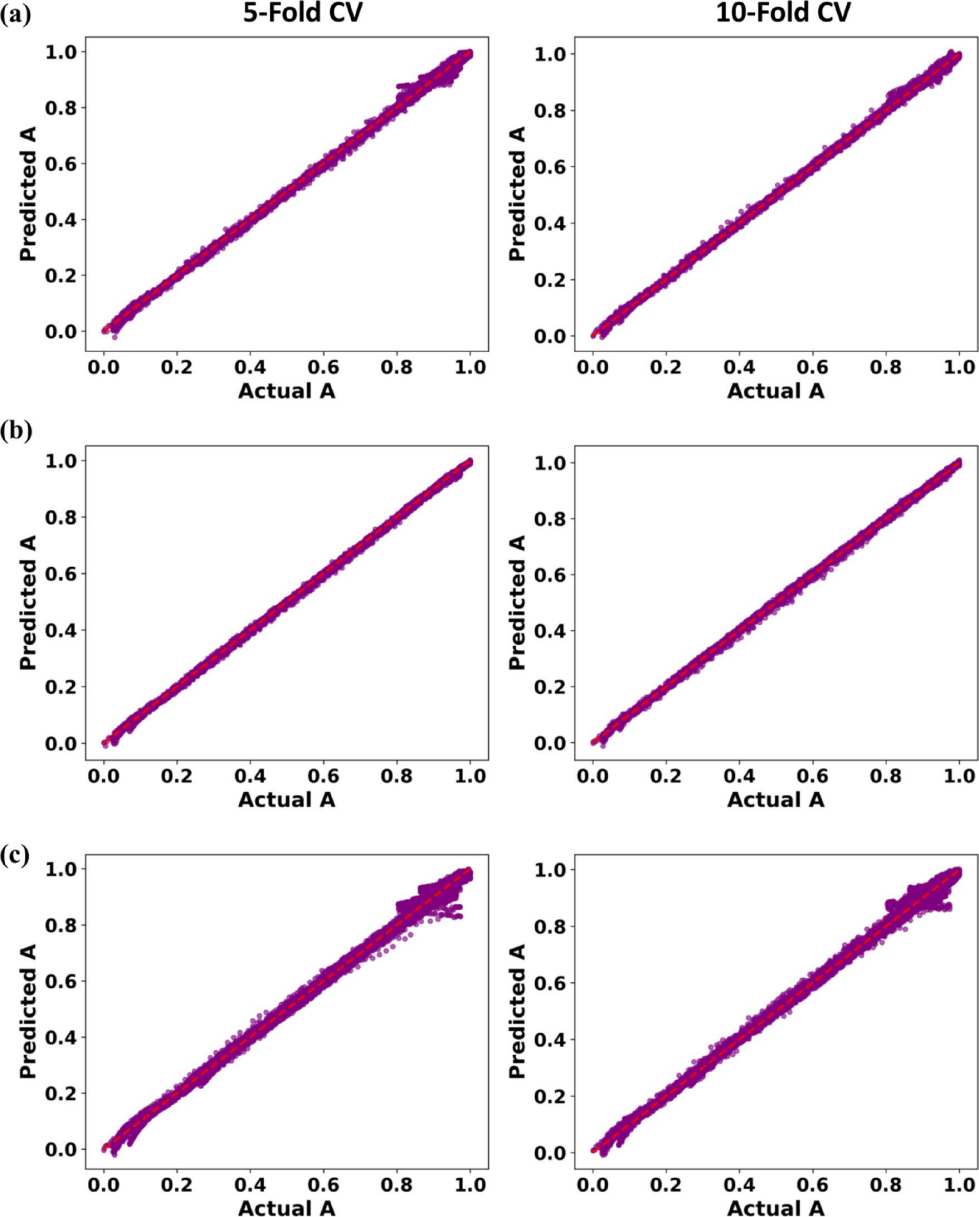
Predicted vs. actual absorptivity for (**a**) MLP, (**b**) Residual MLP, and (**c**) Deep & Cross Net.

**Fig. 10:**
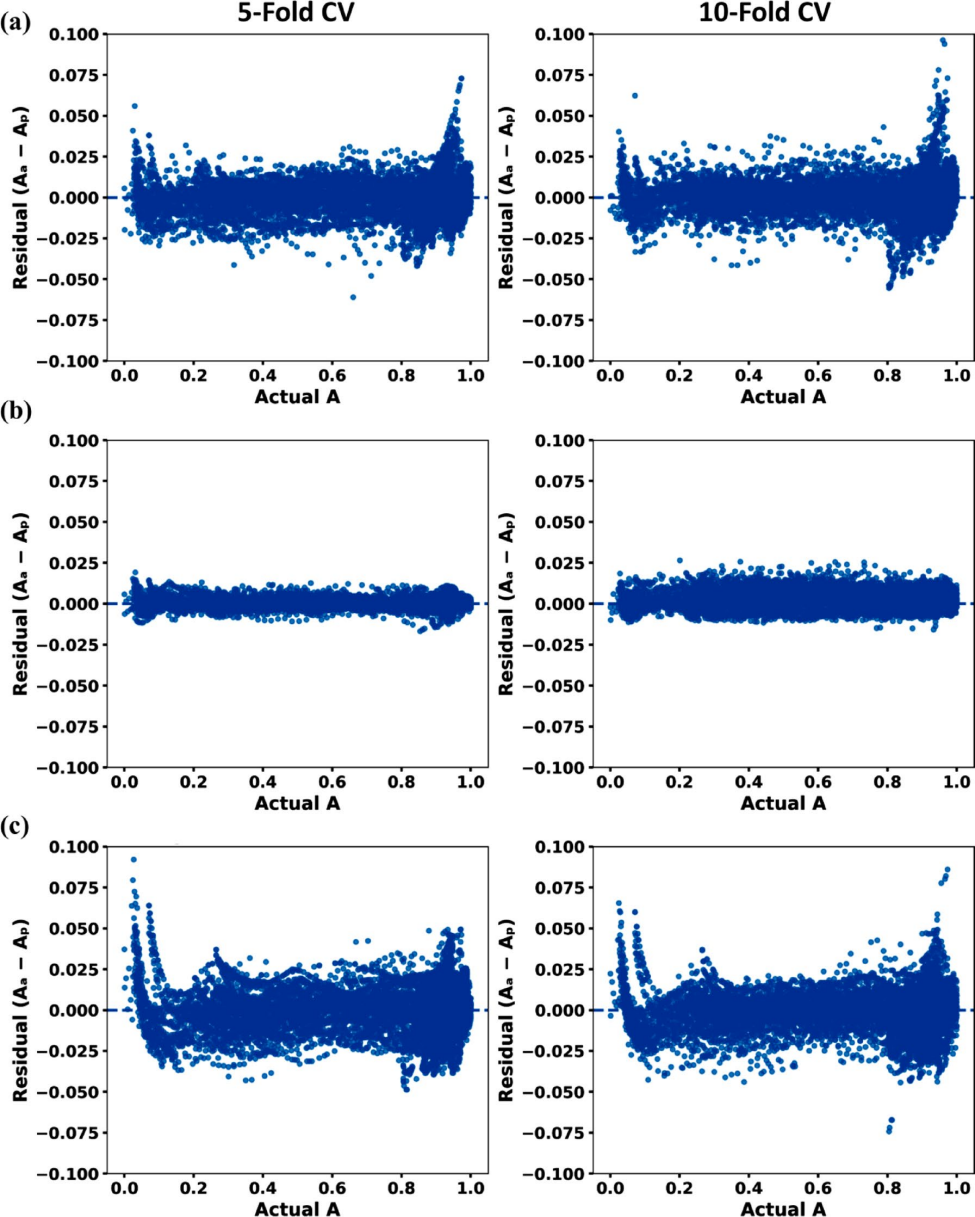
Residual error distribution of the deep learning models: (**a**) MLP, (**b**) R-MLP, and (**c**) Deep & Cross Network, evaluated under 5-fold and 10-fold CV.

**Fig. 11:**
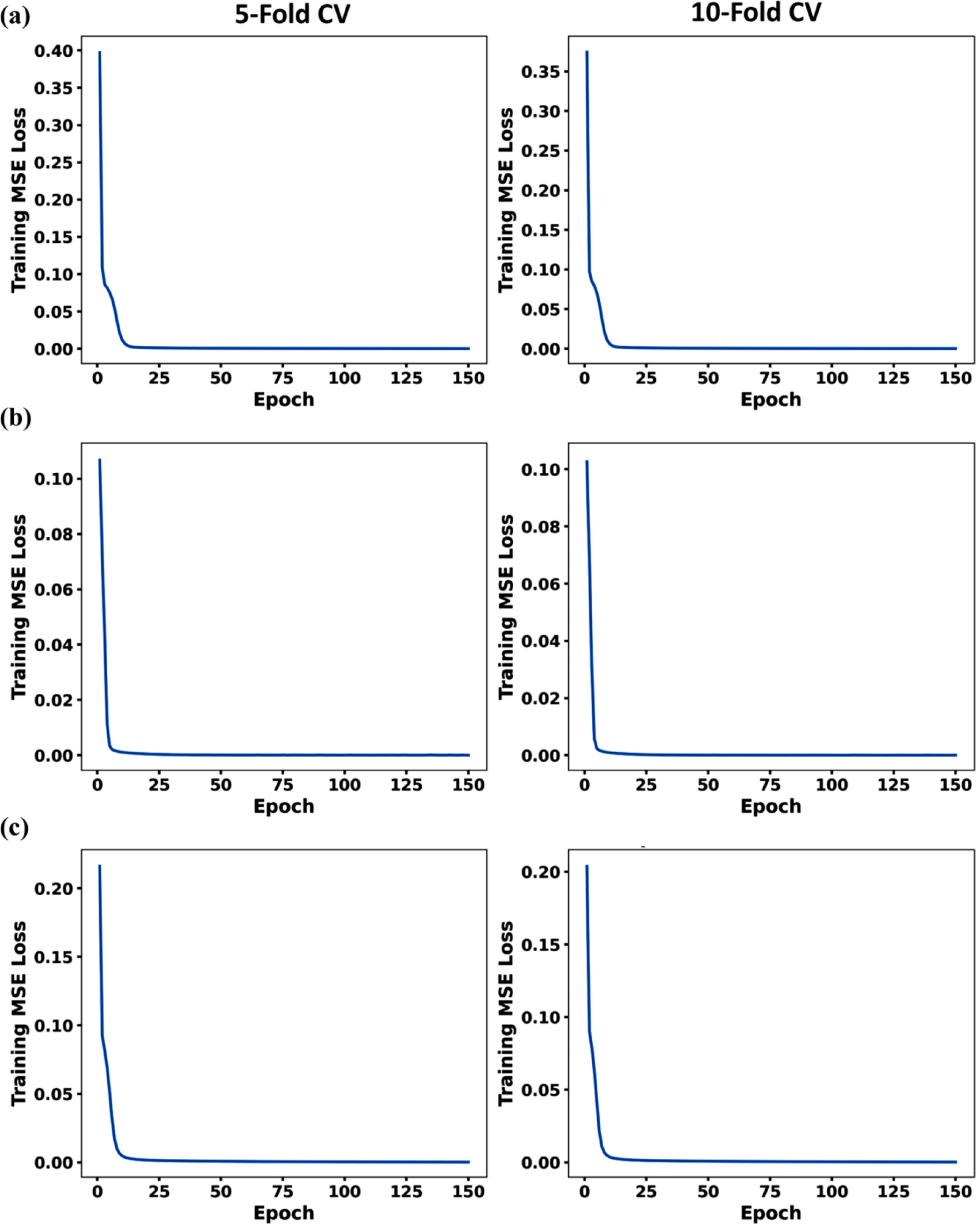
Training convergence behavior of the deep learning models (**a**) MLP, (**b**) R-MLP, and (**c**) DCN shown as training mean squared error (MSE) loss versus epoch under 5-fold and 10-fold CV.

Figure 9 compares the predicted and actual absorption values obtained from the three deep learning models: (a) MLP, (b) Residual MLP, and (c) Deep & Cross Network, each evaluated under both 5-fold and 10-fold cross-validation. All models show good agreement with the ideal regression line, indicating high predictive accuracy and good generalization. Among them, the Residual MLP (Fig. 9b) exhibits the clustering around the diagonal in both 5 & 10 CV, demonstrating minimal deviation across folds. Figure 9a shows the MLP method also performs well, with only slight dispersion at higher absorption levels, whereas the DCN shows comparatively better scattering near the upper absorption range as shown in Fig. 9c.

Figure 10 presents the residual distributions which shows the prediction error against the actual absorptivity. The MLP residuals remain centred around zero but become more scattered at higher absorptivity values (A ≳ 0.8), which is expected near sharp resonance regions, as seen in Fig. 10a. The R-MLP shows compact and uniformly spread residuals over the full absorption range for both CV, as shown in Fig. 10b. The DCN exhibits wider residual dispersion at very low and very high absorptivity values, as illustrated in Fig. 10c. This occurs because the dataset contains fewer samples in these extreme regions (A < 0.1 and A > 0.95), and the DCN’s multiplicative feature-interaction mechanism amplifies small variations in sparsely sampled zones, producing the noticeable tails in Fig. 10c.

Figure 11 depicts the training loss evolution, providing insight into optimization stability and convergence behaviour. The training MSE decreases rapidly during the first few epochs and then converges smoothly to a stable minimum, indicating effective optimization. The MLP takes slightly more epochs to stabilize, whereas the R-MLP achieves the fastest and smoothest convergence, highlighting the importance of residual connections in improving training stability, as shown in Fig. 11a and b. The DCN also converges efficiently, although with minor early-stage fluctuations caused by its increased architectural complexity, as shown in Fig. 11c.

**Table 3:** Mean and standard deviation of R^2^, MSE, and MAE.

Model	CV Scheme	R^2^ (mean ± SD)	MSE (mean ± SD)	MAE (mean ± SD)
MLP	5-Fold	0.9976 ± 0.0006	0.000194 ± 0.000052	0.0098 ± 0.0010
	10-Fold	0.9978 ± 0.0010	0.000177 ± 0.000083	0.0094 ± 0.0024
Residual MLP	5-Fold	0.9996 ± 0.0003	0.000030 ± 0.000024	0.0039 ± 0.0014
	10-Fold	0.9995 ± 0.0005	0.000044 ± 0.000043	0.0050 ± 0.0029
Deep & Cross Net	5-Fold	0.9969 ± 0.0007	0.000257 ± 0.000062	0.0113 ± 0.0011
	10-Fold	0.9971 ± 0.0007	0.000238 ± 0.000053	0.0107 ± 0.0011

**Fig. 12:**
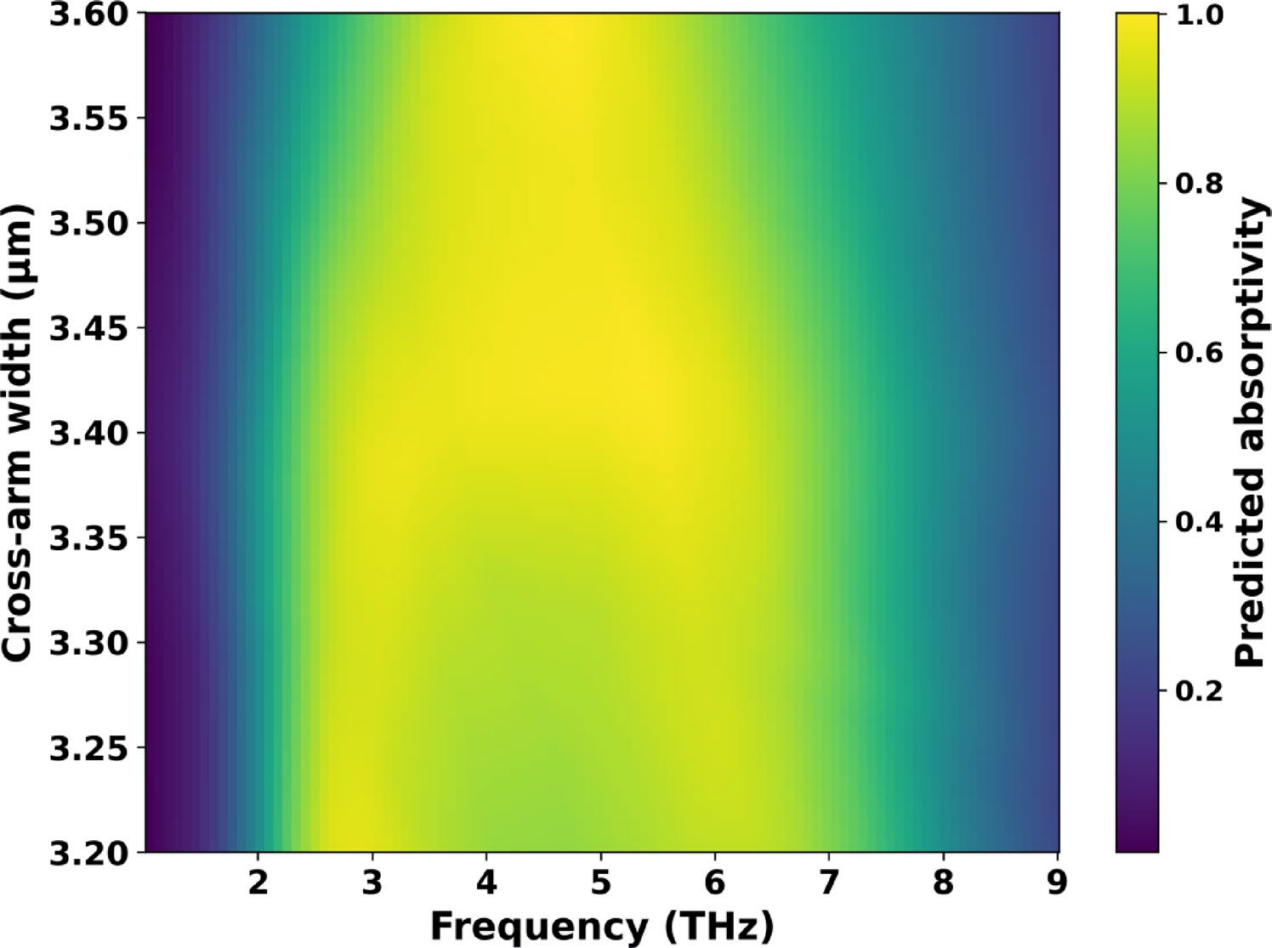
Heat map of the predicted absorptivity as a function of frequency and cross-arm width *d*, generated using the Residual MLP model.

For a quantitative assessment of model performance, Table 3 reports the mean and standard deviation of R^2^, MSE, and MAE under both 5-fold and 10-fold CV. The Residual MLP clearly outperforms the other models, achieving the highest R^2^ values (up to 0.9996) and simultaneously maintaining the lowest MSE and MAE across both CV schemes. In addition, a deep-learning-based heat map was generated using the Residual MLP model to visualize the predicted absorptivity as a function of frequency and cross-arm width, with the remaining parameters held fixed as shown in Fig. 12.

## Conclusions

This study presents a compact broadband MMA that achieves near-perfect absorption across 2.66–6.48 THz under normal incidence. The absorption mechanism is explained through impedance-matching and EM field analyses at multiple resonant frequencies. Parametric studies confirm the structural robustness, while polarization and angular stability analyses verify polarization-insensitive performance for polarization angles up to 90°. Deep learning models evaluated using 5-fold and 10-fold CV help estimate the absorption characteristics and reduce the need for repeated simulation sweeps. The integration of absorber design with DL based prediction provides an efficient and reliable workflow for broadband MMA development. Due to its compact geometry, wide operational bandwidth, and VO_2_-enabled tunability, the proposed MMA holds strong potential for practical implementation in terahertz imaging, stealth technologies, sensing, and THz modulation systems.

## Data Availability

All data that support the findings of this study are available from the corresponding author upon reasonable request.
